# Disinhibition-Induced Delayed Onset of Epileptic Spike-Wave Discharges in a Five Variable Model of Cortex and Thalamus

**DOI:** 10.3389/fncom.2016.00028

**Published:** 2016-04-05

**Authors:** Suyu Liu, Qingyun Wang, Denggui Fan

**Affiliations:** Department of Dynamics and Control, Beihang UniversityBeijing, China

**Keywords:** absence seizures, spike-wave discharges, disinhibitory input, delay onset, bifurcation

## Abstract

Based on a modified neural field network model composed of cortex and thalamus, we here propose a computational framework to investigate the onset control of absence seizure, which is characterized by the spike-wave discharges. Firstly, we briefly demonstrate the existence of various transition types in Taylor's model by increasing the thalamic input. Furthermore, after the disinhibitory function is reasonably introduced into the Taylor's model, we can observe the occurrence of various transition states of firing patterns with different dominant frequencies as the thalamic input is varied under different disinhibitory effects onto the pyramidal neural population. Interestingly, it is found that the onset of spike-wave discharges can be delayed as the disinhibitory input is considered. More importantly, we explore bifurcation mechanism of firing transitions as some key parameters are changed. And also, we observe other dynamical states, such as simple oscillations and saturated discharges with different spatial scales, which are consistent with previous theoretical or experimental findings.

## 1. Introduction

Absence seizure in epilepsy is a chronic neurological disorder in human brain. Pathological manifestations of absence seizure are mainly involved with the generalized abrupt and transient abnormal discharge of neural populations within the brain. Patients with absence seizures are poorly treated by current medications and the patients' quality of life cannot yet be significantly improved (Blumenfeld, 2005; Witcher and Ellis, 2012). Morphologically, absence seizure can be characterized by the typically generalized 2–4 Hz spike-wave discharge (SWD) and transition of some typical firing states (Steriade and Contreras, 1998; Crunelli and Leresche, 2002; Coenen and van Luijtelaar, 2003; Budde et al., 2005), which has been captured from the electroencephalogram (EEG) of individual patients during the epileptic absence seizures. However, the mechanisms underlying the SWD and its transition of absence seizure are still unknown, and further exploration is very necessary.

There are many developed models for investigating internal properties of epileptic seizure such as neuronal network (Reato et al., 2012; Naze et al., 2015), mean fields (van Albada and Robinson, 2009; van Albada et al., 2009; Hu et al., 2015) and neural fields (Taylor et al., 2013a,b, 2014, 2015; González-Ramírez et al., 2015), by which, the epileptic seizure can be understood well. In particular, a network model of spiking neurons was proposed to systematically investigate the conditions, under which the network displays the emergent dynamic epileptic behaviors (Naze et al., 2015). Based on the basal ganglia-thalamocortical mean field network model, Chen et al. (2014) set up a computational evidence for absence seizure and demonstrated that the thalamus plays a key role in the bidirectional modulation for the SWD of absence seizures. Neural field models are increasingly improved to mathematically understand the evolution behaviors of epileptic seizures, especially for the SWD of epileptic absence seizure (Taylor and Baier, 2011; Taylor et al., 2013a,b). In particular, following the work of Wendling et al. (2002), Taylor and Baier (2011) proposed a modified model by introducing a second inhibitory variable within the cortex, and they spatially and macroscopically expanded this improved model to investigate the generation mechanisms of SWD during epileptic seizures. However, they are independent of the inhibitory behavior between inhibitory neural populations. Fan et al. (2015) computationally described the epileptic two-way transitions between absence seizures and seizures of tonic-clonic type, which occurs onto the cortical cortex by introducing disinhibitory neuronal populations based on clinical observations (Mayville et al., 2000; Shih and Hirsch, 2003). Based on it, this work also mainly focuses on the role of established competing inhibitory effects in neuronal population of cortex.

**Figure 1:**
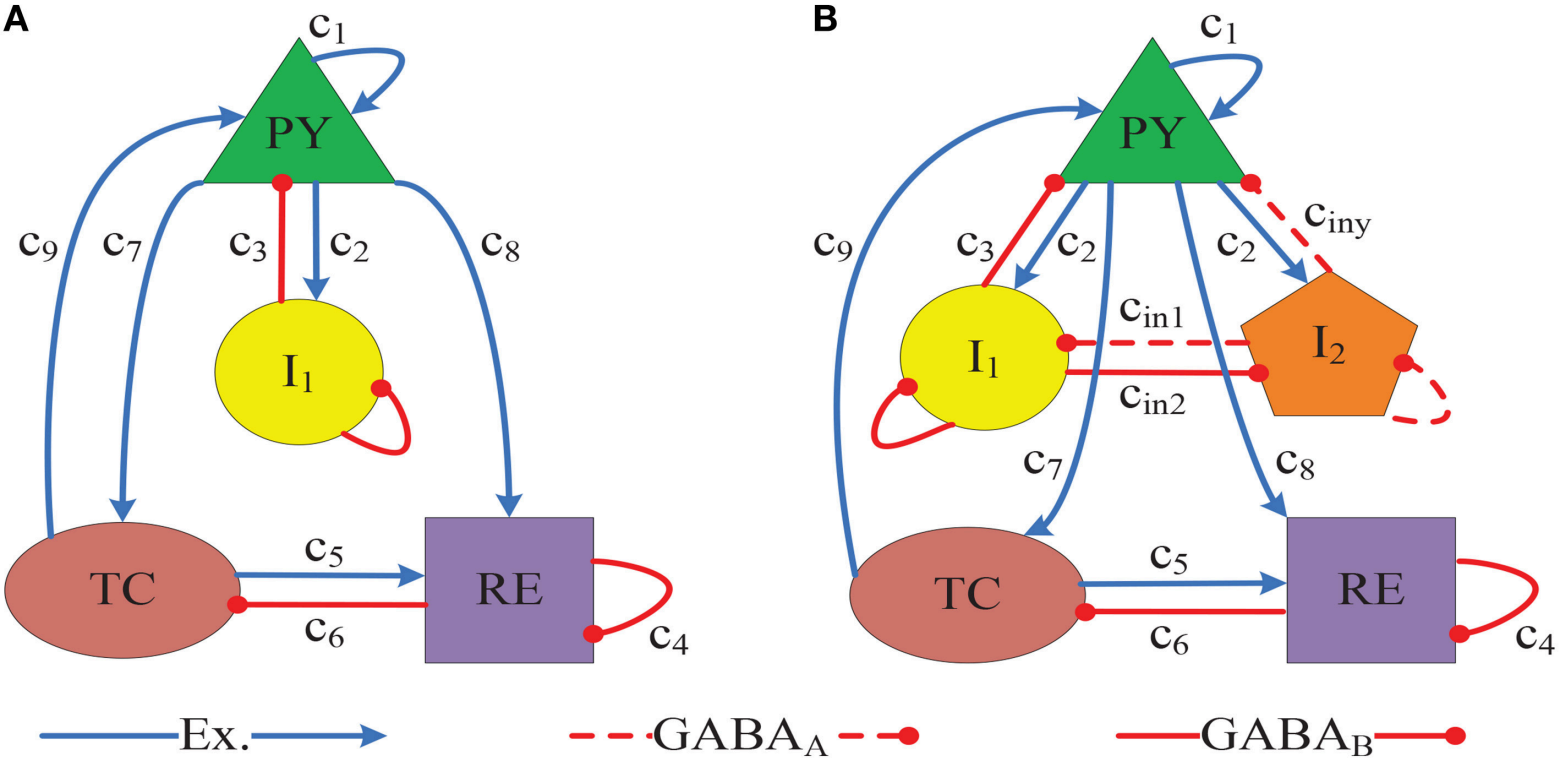
**(A)** Connectivity schematic diagram of the Taylor's thalamocortical model network, which is composed of the cortical subnetwork composed of the pyramidal neural population PY, the interneuronal population *I*_1_, and the subcortical TC-RE subnetwork (Taylor et al., 2015). **(B)** The proposed Taylor' model network adapted from the Taylor's models (Taylor et al., 2013b, 2015) and the modified model of Fan et al. (2015), with the introducing of a second inhibitory neuronal population, *I*_2_, on a different time scale. Blue lines with arrows denote the excitatory projections mediated by glutamate receptors. Red solid (dashed) lines with round heads represent the inhibitory projections mediated by the *GABA*_*B*_ (*GABA*_*A*_) receptors. The used parameter values in this paper are set as follows: *c*_1_ = 1.8, *c*_2_ = 4, *c*_3_ = 1.5, *c*_4_ = 0.2, *c*_5_ = 10.5, *c*_6_ = 0.6, *c*_7_ = 3, *c*_8_ = 3, *c*_9_ = 1, τ_1_ = 26, τ_2_ = 32.5, τ_3_ = 300, τ_4_ = 2.6, τ_5_ = 2.6, *c*_*in*1_ = 0.1, *c*_*in*2_ = 0.1, *h*_*py*_ = −0.35, *h*_*in*1_ = −3.4, *h*_*re*_ = −5, *h*_*in*2_ = −1.50, ε = 100000, *a* = 2.8, *b* = 0.5. And, *c*_*iny*_ and *h*_*tc*_ are two considered main parameters of present paper.

By incorporating brain connectivity derived from magnetic resonance imaging of a subject with idiopathic generalized epilepsy, Taylor et al. (2015) developed a thalamocortical computational model of epileptic SWD dynamics model (see Figure 1A) to evaluate the effectiveness of a pseudospectral method for the simulated seizure abatement. They found that time-varying stimuli generated by the pseudospectral method can successfully abate the simulated seizures. In addition, mounting experimental evidences suggest the important roles for both the cortical cortex and thalamus in the genesis and propagation of epileptic SWD oscillations (Prevett et al., 1995; Seidenbecher et al., 1998; Cope et al., 2009; Volman et al., 2012; Pi et al., 2013). Although the disinhibitory controls performed by the inhibitory interneurons were employed in the model of Fan et al. (2015), they ignored the thalamic function during epileptic absence seizure. Recently, Chen et al. (2014) and Taylor et al. (2013a, 2014, 2015) paid attention on the thalamic mechanisms and obtained some better insights into absence seizures when they mathematically modeled the epileptic seizures. Nevertheless, they overlooked the disinhibitory effects of interneurons within cortical cortex. To deeply understand the mechanisms of SWD for the epileptic absence seizures, we will set up a more complete and biological model (see Figure 1B) by introducing the second inhibitory neuronal populations *I*_2_ with a different time scale into the model of Taylor et al. (2015), and simultaneously consider both the disinhibition of interneuronal population within the cerebral cortex and the subcortical thalamic function. This modified model is independent of the seizure type, and with the addition of a second inhibitory neuron population, the epileptic SWD dynamics can be potentially changed due to the disinhibition modulation.

In addition, effective mathematical models can also demonstrate the dynamical transition of SWD among different dynamical states as some key parameters are changed. For example, Taylor and Baier (2011) discussed a dynamical transition from SWD to 2SWD (two-spike wave discharge) in a space-independent model and the spatially-extended network model, respectively. Chen et al. (2014) showed four different dynamical states (low firing, simple oscillation, SWD, and saturated firing) with different transition mechanisms to obtain the control regions of the absence seizure-free. Furthermore, rich dynamical transition states can be found in the work of Fan et al. (2015). However, corresponding dynamical bifurcation analysis of different states transitions was poorly provided. Biologically, dynamical bifurcation analysis of neuroscience can ideally provide rich insights into the highly spatiotemporally changing brain microenvironment, which can enable us to visualize sudden state changes in dynamical systems under modest changes (Terman, 1992; Izhikevich, 2002; Marten et al., 2009; Gu et al., 2014). Fortunately, Taylor et al. (2015) dynamically analyzed the evolution of epileptic SWD oscillation and they found that a fold of cycles bifurcation can occur between stable focus and SWD oscillations, which can physiologically represent the onset of absence seizure in epilepsy originated from the saturated low firing. Also, another subcritical Hopf bifurcation can take place with the disappearance of stable focus describing the dynamical transitions from simple oscillations to saturated high firing, which biologically represents the transition from clonic seizures of epilepsy to the offset of epileptic seizures.

Presently, we further extend existing results to probe into SWD dynamics of epileptic absence seizures using the modified model based on the work of Taylor et al. (2015). In particular, we propose the computational model of modified thalamocortical network dynamics by introducing a disinhibition neural population. And then, we find that the proposed computational network model can exhibit various transition states of firing patterns with different dominant frequencies as the thalamic input changes with different inhibitory effects onto the pyramidal neural population. Importantly, it is shown that the onset of spike-wave discharges can increasingly be delayed as the disinhibitory input into the pyramidal neural population is increased, and we also study bifurcation mechanisms of firing transitions as some key parameters are changed.

## 2. Description of models

Experimental findings (Destexhe et al., 1994, 1998) have shown that synaptic interactions in the thalamic nucleus are mediated by glutamatergic and GABAergic receptors using kinetic models of postsynaptic receptors, namely possible involvement of AMPA EPSPs (*TC* → *RE*) (Bhattacharya, 2013), and *GABA*_*A*_/*GABA*_*B*_ IPSPs (*RE* → *TC*) (Destexhe et al., 1999) which mediate firing operating on two different inhibitory time scales. Inspired by this finding, a thalamocortical model (see Figure 1A) has been developed, which includes cortical excitatory pyramidal(PY) cells and inhibitory interneurons (*I*_1_), as well as the subcortical TC-RE circuit model composed of the thalamocortical relay cells (TC) neurons and neurons located in the reticular nucleus(RE), as described in Taylor et al. (2015) where their established model is regarded as the Taylor's model here. However, recent experimental result (Pi et al., 2013) has shown that there exists a basic disinhibitory circuit module in the mammalian cerebral cortex, which means that there are mutual effects among different inhibitory neurons with different time scales. Hence, there are the important disinhibitory modulation functions for the cortical epileptic dynamics. Motivated by this, we presently develop a modified model (see Figure 1B) with the addition of a second inhibitory neuronal population, *I*_2_, to the Taylor's model. Resultant governing equations can be described as follows,

(1) $$\begin{array}{l} \frac{dPY(t)}{dt}=\tau_{1}(h_{py}-PY+c_{1}f(PY)-c_{3}f(I_{1})\\ \;+\;c_{9}f(TC)-c_{iny}f(I_{2})) \end{array}$$

(2) $$\begin{array}{rcl} \frac{dI_{1}\left(t\right)}{dt} & = & \tau_{2}\left(h_{in1}-I_{1}+c_{2}f\left(PY\right)-c_{in1}f\left(I_{2}\right)\right) \end{array}$$

(3) $$\begin{array}{rcl} \frac{dI_{2}\left(t\right)}{dt} & = & \tau_{3}\left(h_{in2}-I_{2}+c_{2}f\left(PY\right)-c_{in2}f\left(I_{1}\right)\right) \end{array}$$

(4) $$\begin{array}{rcl} \frac{dTC\left(t\right)}{dt} & = & \tau_{4}\left(h_{tc}-TC-c_{6}g\left(RE\right)+c_{7}f\left(PY\right)\right) \end{array}$$

(5) $$\begin{array}{rcl} \frac{dRE\left(t\right)}{dt} & = & \tau_{5}\left(h_{re}-RE-c_{4}g\left(RE\right)+c_{5}g\left(TC\right)+c_{8}f\left(PY\right)\right). \end{array}$$

where *I*_1_ and *I*_2_ represent the two different types of inhibitory neural populations with two different slow and fast time scales, which are determined by the parameters τ_2_ and τ_3_, respectively. *h*_*py, in*1, *in*2, *tc, re*_ are input parameters indicating the additive constants as used in the model of Taylor and Baier (2011), *c*_1, …, 9, *iny, in*1, *in*2_ are the connectivity parameters within different neural populations, τ_1, 2, 3, 4, 5_ are time scale parameters.

In particular, *f*(*x*) = 1/(1+ε^−*x*^) and *g*(*y*) = *ay*+*b* are transfer functions as used in models (Taylor et al., 2014, 2015), and multiplied by a connectivity parameter *c*_1, …, 9, *cin*1, *cin*2, *ciny*_ which together mediate input from one population to another severally. Thereinto, ε and *a* determine the steepness of the two transfer functions respectively, and *x* = *PY, I*_1_ and *I*_2_, and *y* = *TC* and *RE*. The sigmoid one is employed by cortical variables and thalamic TC population activating the pyramidal population, and the linear one is used for the thalamic subsystem instead of the sigmoid function *f*(*x*) to qualitatively mirror the dynamics of epileptic seizure and make simple analysis. In addition, unless noted otherwise, simulating parameter values follow the previous works (Taylor and Baier, 2011; Taylor et al., 2015).

## 3. Results

We first modify the Taylor's model (see Figure 1A) by introducing the second inhibitory neural population. And then, we investigate transition dynamics of the modified Taylor's model, which can be related to the epileptic seizures. Importantly, the spatio-temporal evolution dynamics of epileptic SWD oscillation will be indeed explored as the disinhibition effect induced by two inhibitory populations *I*_1_ and *I*_2_ (see Figure 1B) is introduced.

### 3.1. Transition dynamics in the Taylor's model

Because of the essential effect of thalamic activities on the epileptic seizure activities, an external thalamic input, *h*_*tc*_, can be seen as the bifurcation parameter to explore the transition dynamics of epileptic SWD oscillations, by controlling the excitatory effect of thalamus on the cortical subnetwork. Generally, *h*_*tc*_ is set to the negative values. Local extrema (maximums and minimums) of the resulting numerical time series for the PY neural field potentials are plotted to show firing behaviors of neural population as the parameter changes.

**Figure 2:**
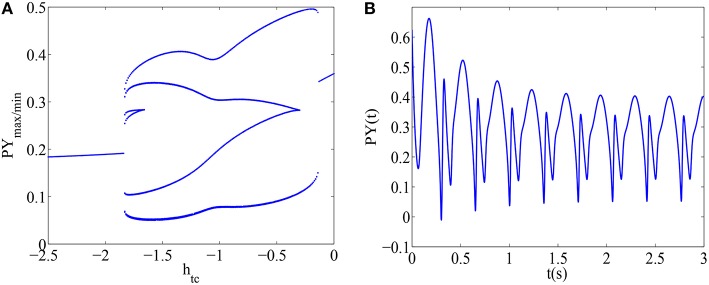
**(A)** Bifurcation diagram of PY in the original thalamocortical model developed by Taylor et al. is shown as the parameter *h*_*tc*_ varies; **(B)** A typical epileptic wave seizure is illustrated as the parameter *h*_*tc*_ = −1.5. All other parameters are given as the above.

Bifurcation diagram of PY in the original thalamocortical model developed by Taylor et al. (2015) with respect to *h*_*tc*_ is shown in Figure 2A. From Figure 2A, we can observe rich dynamical transitions as the bifurcation parameter *h*_*tc*_ increases in interval (−2.5, 0), i.e., gradually decreasing inhibition function on the thalamus, which can imply the increase of the thalamic excitability. Firstly, for smaller value of *h*_*tc*_, the excitability of thalamus can be inhibited. Hence, little thalamic excitatory input can be received by the cortex which leads to the saturated firing of PY. Thereafter, as the value of *h*_*tc*_ is increased, the Taylor's model can display a dynamical transition from periodic 2-spike and wave discharges, periodic 1-spike and wave discharges (SWD) to simple oscillations. As typical case for the SWD, which is the main hallmark of epileptic absence seizures, the corresponding time series of epileptic SWD oscillation can be seen in Figure 2B with *h*_*tc*_ = −1.5. Moreover, as the value of *h*_*tc*_ is approaching to zero, i.e., external inhibitory functions on the thalamus disappear, the dynamics of the Taylor's model will be restored to the saturated firing with a different scale.

In short, the Taylor's model bears rich transition dynamics as variable *h*_*tc*_ increases. This is deliberately decreasing inhibition function on thalamus, which is shown as the steady-state firing pattern change of PY population from saturated firing, 2-periodic spike and wave discharges, SWD, simple oscillations to saturated firing with a different scale with attenuating external inhibitory functions on the thalamus.

### 3.2. Transition dynamics in the modified Taylor's model

**Figure 3:**
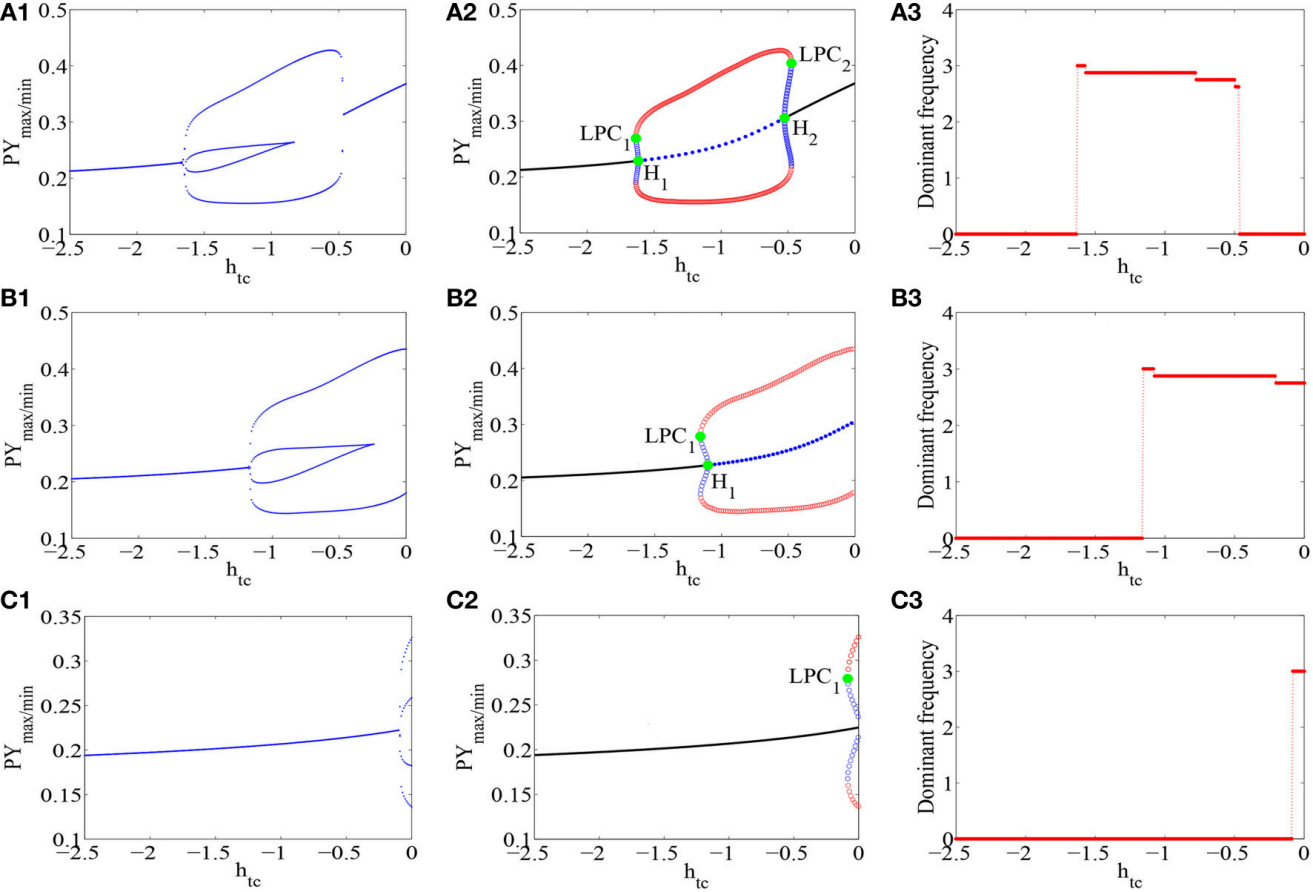
**The bifurcation diagrams of PY neural field potentials (A_1_,B_1_,C_1_), and their corresponding dynamical analysis (A_2_,B_2_,C_2_) as well as the dominant frequencies (A_3_,B_3_,C_3_) are shown for the improved Taylor's model as the thalamic inputs, *h*_*tc*_, varies, with *c*_*iny*_ = 0.005 (A_1_–A_3_), *c*_*iny*_ = 0.05 (B_1_–B_3_) and *c*_*iny*_ = 0.15 (C_1_–C_3_), respectively**.

With introducing the second inhibitory neural population *I*_2_ into the Taylor's model, dynamics of the epileptic SWD oscillations in the improved model can be significantly influenced by the disinhibition of the second inhibitory neural population *I*_2_ on the first one *I*_1_ and disinhibitory effects onto the pyramidal neural population. In particular, compared Figure 3A_1_ with Figure 2A, we can find that 2-periodic spike and slow-wave discharges disappear due to the interactions of neural populations in the modified Taylor's model. Thus, the stable 1-periodic spike with slow-wave discharges and simple oscillations are still kept. Further investigation shows that amplitudes of SWD oscillation in modified Taylor's model are smaller than those in Taylor's model, and parameter ranges for the SWD oscillation also become small. As shown in Figure 3A_1_, there are stable 1-periodic spike with slow-wave discharges in the interval of parameter *h*_*tc*_ ~∈ [−1.636, −0.88], and the simple oscillation is located in the interval *h*_*tc*_ ~∈ [−0.88, −0.5259].

Underlying bifurcation mechanisms are very helpful to understand these transitions of the disinhibition induced oscillations. To do this, the corresponding bifurcation diagram is given in Figure 3B_1_). It can be found that for the smaller or larger values of *h*_*tc*_, the fixed points can be observed, which corresponds to the saturated discharges of neural populations with different spatial scales, respectively. When *h*_*tc*_ ≈ −1.636, one stable and one unstable limit cycles occurring around the stable focus are produced by fold limit cycle bifurcation (*LPC*_1_). Resultantly, a bistable region appears with the coexistence of stable limit cycle and unstable one. Hence, the dynamics of the modified system can transit from the saturated firings to the periodic 1-spike and slow-wave discharge. In addition, it can be seen that as *h*_*tc*_ is further increased, the focus undergoes a subcritical Hopf bifurcation (*H*_1_) at *h*_*tc*_ ≈ −1.618, which ends the boundary of bistable region, and leads to the instability of focus immediately. Resultantly, the system successively immerses in the periodic SWD oscillation. With the further increasing *h*_*tc*_, another subcritical Hopf bifurcation (*H*_2_) takes place, where the unstable focus becomes stable and an unstable limit cycle occurs, hence forming another bistable region. As *h*_*tc*_ becomes a bit larger, the stable limit cycle and the unstable one annihilate each other through an another fold limit cycle bifurcation (*LPC*_2_). And the system finally transits into the saturated firing again.

All in all, employing bifurcation analysis potentially uncovers the transition mechanisms of oscillatory patterns (SWD, simple oscillation and saturated firing) as key parameter *h*_*tc*_ is changed in the modified Taylor's model. Compared to the Taylor's model, this can provide rich insights into the spatiotemporally changing brain microenvironment, which can enable one to visualize sudden state changes in dynamical systems as the model parameters are changed.

### 3.3. The delay control of spike-wave dynamics for the modified Taylor's model

As discussed above, as the effect of disinhibitory neural population *I*_2_ is involved in the modified model, rich dynamics of the thalamocortical model can be dramatically affected with some key parameters being changed. Interestingly, we can find from Figure 3A_1_ that the onset of absence seizure in the modified model can be delayed as disinhibitory neural population is introduced. To clearly investigate this fact, we will in detail provide the bifurcation diagrams of PY neural field potentials, and their corresponding dynamical bifurcation analysis as well as the dominant frequencies as *h*_*tc*_ varies. In particular, we choose three typical values of disinhibitory effects onto the pyramidal neural population *c*_*iny*_, the results are depicted as shown in Figure 3. Initially, we can observe from Figures 3A_1_,B_1_,C_1_ that the onset of SWD is postponed as *h*_*tc*_ changes for different disinhibitory input into the pyramidal neural population *c*_*iny*_. Hence, it can be concluded that disinhibitory neural population can prolong bifurcation of the equilibrium state to oscillation state. For more details, bifurcation analysis from Figures 3A_2_,B_2_,C_2_ show that as the parameter *h*_*tc*_ changes, the modified model transits to SWD oscillations via fold bifurcation of limit cycles (LPC), which goes through the coexisting region of an unstable and stable limit cycles. In addition, as the disinhibitory input into the pyramidal neural population *c*_*iny*_ becomes large, SWD oscillation state can appear with the large *h*_*tc*_. And also, the dominant frequencies that correspond to the state bifurcation diagrams are shown in Figures 3A_3_,B_3_,C_3_, from which we can quantify the frequencies of SWD and simple oscillations. It is obvious that the frequencies of SWD are located within the typical 2–4 Hz. Biologically, this represents the absence seizures and clonic seizures.

**Figure 4:**
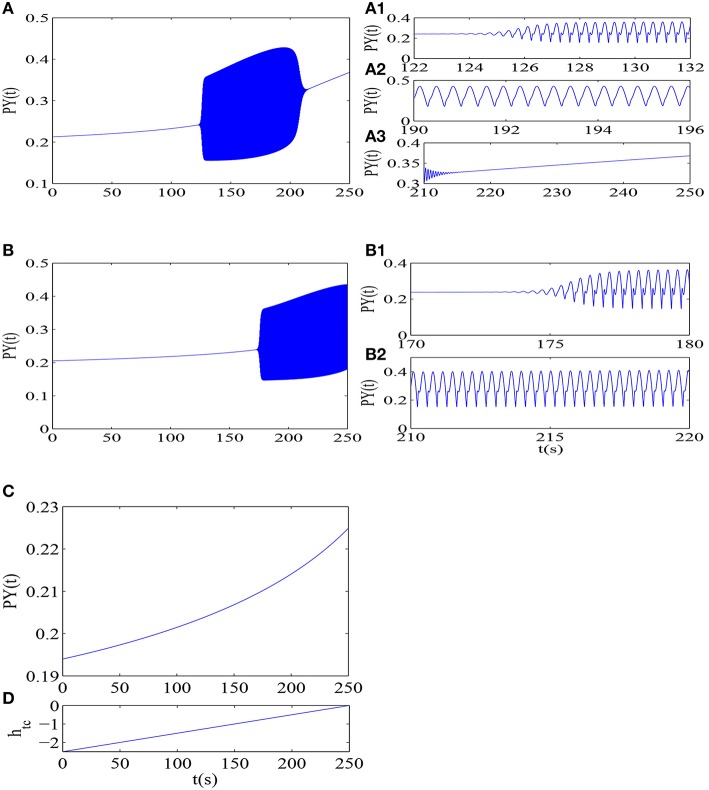
**The time series for the different values of the disinhibitory input into the pyramidal neural population *c*_*iny*_: (A) *c*_*iny*_ = 0.005, (B) *c*_*iny*_ = 0.05, and (C) *c*_*iny*_ = 0.15**. This corresponds to the Figure 3 and it is accompanied by linearly increasing of *h*_*tc*_ over [-2.5, 0] with *t* varying in (0, 250 *s*) **(D)**. Here, **(A**_**1**_**–A**_**3**_**)** correspond to the different phases of **(A)**: SWD **(A**_**1**_**)**, simple oscillation **(A**_**2**_**)** and saturated firing **(A**_**3**_**)**, where we can see that the onset of epileptic SWD is around *t* ≈ 126*s*, i.e., *h*_*tc*_ ≈ −1. **(B**_**1**_, **B**_**2**_**)** correspond to the different phases of **(B)**: SWD **(B**_**1**_**)** and simple oscillation **(B**_**2**_**)**, where the onset of epileptic SWD is around *t* ≈ 176*s*, i.e., *h*_*tc*_ ≈ −0.8.

In order to temporally illustrate the delayed onset of epileptic absence seizures and fully understand the results of Figure 3, we first suppose that the external inhibitory thalamic input *h*_*tc*_ is time-dependent, and it linearly increases over the time (see Figure 4D), which results in gradual disinhibition of the thalamus. It is clear from Figure 4A that the system can display different equilibrium states and the epileptic absence seizures characterized by the typical 2–4 Hz SWD as *h*_*tc*_ is linearly increased. And, when *h*_*tc*_ is increased to the large enough value, SWD can occur, which is a good vision on understanding the onset of SWD as shown in Figure 3. Particularly, in the right of the Figure 4A, different phases of Figure 4A that represent various firing states are provided (see Figures 4A_1_–A_3_), where the onset of epileptic SWD is around *t* ≈ 126*s*, i.e., *h*_*tc*_ ≈ –1. However, as the *c*_*iny*_ is set to another larger value, *c*_*iny*_ = 0.05, the onset of epileptic SWD can be delayed to around *t* ≈ 176*s* (Figure 4B), i.e., *h*_*tc*_ ≈ –0.8 (also see Figures 4B_1_,B_2_). In addition, as the *h*_*tc*_ increases to *h*_*tc*_ = 0.15, larger delay for the onset of epileptic SWD can be found as shown in Figure 4C, where SWD can not be seen in the considered time.

**Figure 5:**
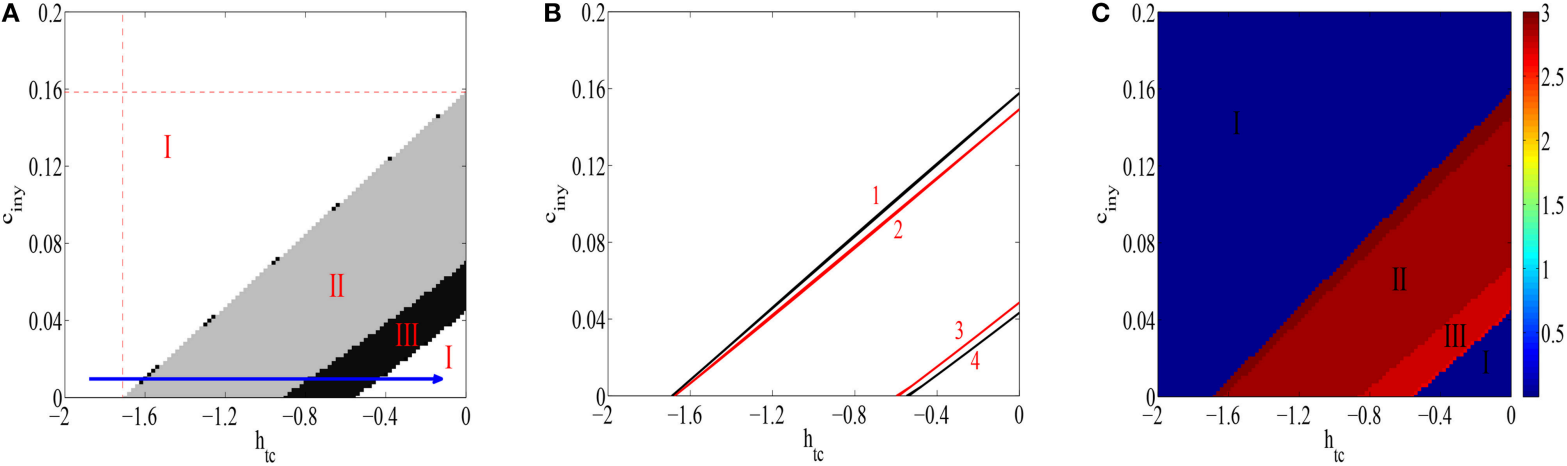
**The different firing states (A), the diagram of dynamical bifurcation analysis (B), and the variations of corresponding dominant frequencies (C), for the improved Taylor's model are exhibited as two parameters *h*_*tc*_ and *c*_*iny*_ are changed in considered regions of the present paper**. In particular, I denotes the saturated firing with different spatial scales, II and III are the SWD and simple oscillations, respectively. It is shown that the red curves represent the Hopf bifurcation curves, and the black curves represent the fold limit cycle bifurcation curves of the limit cycle. And, two narrow areas between black and red curve are the bistable regions, where stable and unstable oscillations can coexist. Additionally, SWD of epileptic absence seizure and epileptic clonic oscillations occurs between two black curves.

Nextly, to observe the overall dynamics transition of the modified Taylor's model and study how transition dynamics can be quantitatively influenced by both thalamic input parameter *h*_*tc*_ and inhibitory strength *c*_*iny*_, we depict the state transitions (Figure 5A), the corresponding dynamical bifurcation analysis (Figure 5B) and the corresponding dominant frequency (Figure 5C) of this modified Taylor's model on the parameter plane of (*h*_*tc*_, *c*_*iny*_) with the region of [−2, 0] × [0, 0.2]. We can see in Figure 5A that there are three types of firing states, which are denoted as: I: saturated firing, II: periodic spike with slow-wave discharges and III: simple oscillations. Furthermore, the detailed bifurcation analysis of this modified model is given in Figure 5B on the parameter plane of (*h*_*tc*_, *c*_*iny*_). Thereinto, the red curves represent the Hopf-type bifurcation curves of the equilibrium points, and the black curves represent the fold limit cycle bifurcation curves of the limit cycles. It is noted that the two narrow areas between black and red curves, i.e., the regions between curves 1 and 2 or curves 3 and 4, are the bistable regions of this system, where stable and unstable oscillations can coexist. Moreover, between two black curves, i.e., curves 1 and 4, SWD of epileptic absence seizure and epileptic clonic oscillations can occur, which corresponds to the regions II and III of Figure 5A. For other regions, stable fixed points representing the saturated firing of the PY neural population can occur (see the region I in Figure 5A). As typical example, the direction of the blue arrow in the Figure 5A indicates the increasing of *h*_*tc*_, i.e., gradual disinhibition on the thalamus, which successively leads to the various state transitions including the SWD of epileptic absence seizures. Particularly, compared Figure 5A with Figure 5B, it is seen that the positive slop of curve 1 determines the delayed onsets of epileptic SWD oscillations as the *c*_*iny*_ increases. This also implies that inhibitory effects can weaken the excitability of thalamus and hence influence the potential dynamical behaviors of the modified Taylor's model. In addition, we can also see from Figure 5A that for the less *h*_*tc*_ (< 0) or larger absolute values of *h*_*tc*_, e.g., *h*_*tc*_ ~< −1.7, the excitability of the thalamus can be effectively inhibited. And then, the dynamics of the cortical subnetwork can less be influenced by the subcortical thalamic excitatory input. Hence, the system reaches to the state of saturated firing. Similarly, for any less *h*_*tc*_ and larger *c*_*iny*_ ~> 0.16 of the considered region in this paper, the system always lies into the saturated firing state, which implies that *c*_*iny*_ can counteract the disinhibition function of *h*_*tc*_ on the thalamus.

**Figure 6:**
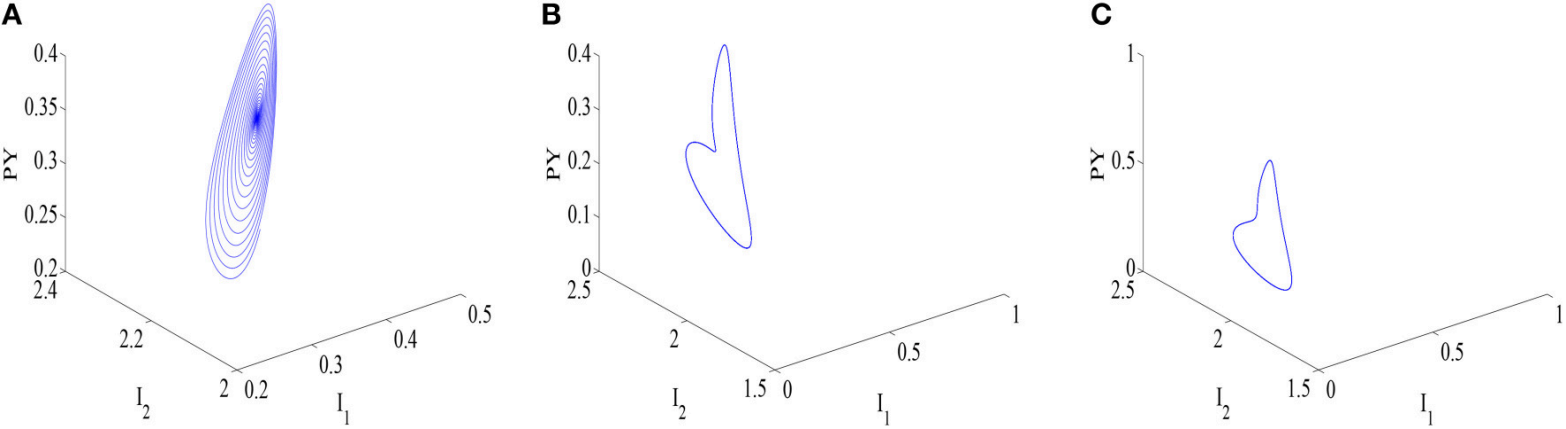
**The phase space plots of the subsystem *PY*−*I*_1_−*I*_2_ corresponding to the Figure 5A: (A)** equilibrium point with *h*_*tc*_ = −0.25 and *c*_*iny*_ = 0.02, **(B)** limit cycle representing SWD of epileptic absence seizures with *h*_*tc*_ = −0.5, *c*_*iny*_ = 0.05, and the **(C)** limit cycle representing simple oscillation with *h*_*tc*_ = −0.25 and *c*_*iny*_ = 0.05.

**Figure 7:**
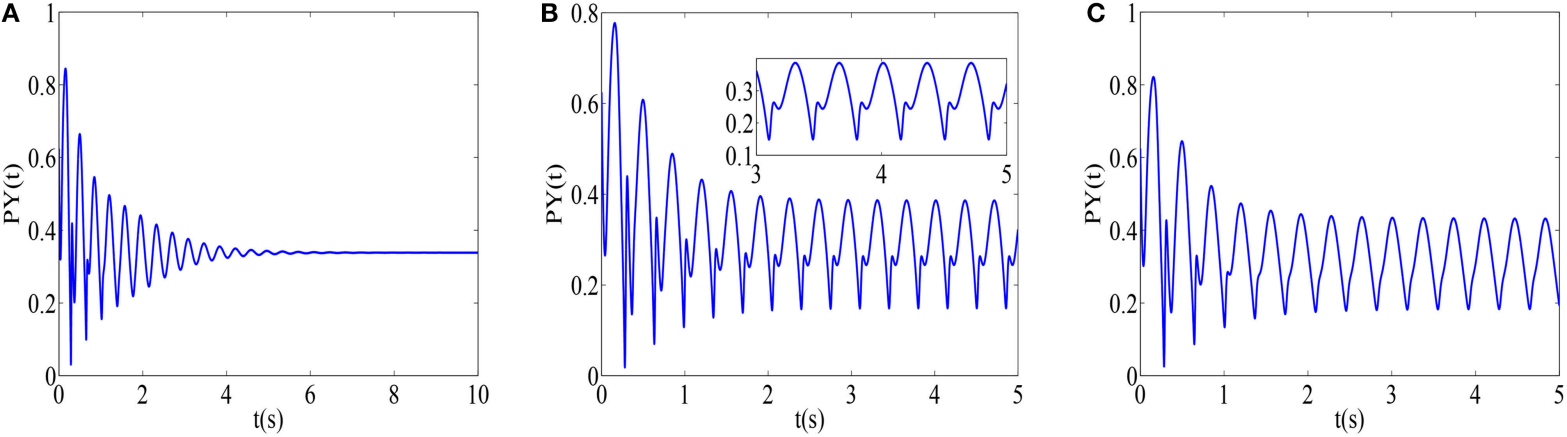
**The time series on the different firing states corresponding to the Figure 6: (A)** saturated firing with *h*_*tc*_ = −0.25 and *c*_*iny*_ = 0.02, **(B)** SWD (see the inset) of epileptic absence seizures with *h*_*tc*_ = −0.5, *c*_*iny*_ = 0.05 and the **(C)** simple oscillation of epileptic clonic seizures with *h*_*tc*_ = −0.25 and *c*_*iny*_ = 0.05.

For the better explanation of disinhibition mechanisms, as an illustration, typical parameters are chosen on the three different state regions (I, II, III), and the corresponding phase diagrams of variables (*PY, I*_1_, *I*_2_) are shown in Figures 6A–C, respectively. The trajectory in the *PY* − *I*_1_ − *I*_2_ space in Figure 6A appears to embrace the equilibrium point. Furthermore, a limit cycle attractor with sharp point representing epileptic absence seizures emerges in Figure 6B, which is comparable to the ones of Figure 6C with the difference of parameter *h*_*tc*_. The further increasing *c*_*iny*_ can lead to a limit cycle attractor in Figure 6C, which represents the simple oscillation of the modified model. Additionally, according to the oscillatory frequency (Figure 5C), firing states II and III can be considered as the epileptic absence seizures and clonic seizures, and the time series corresponding to Figures 6A–C, respectively are shown in Figures 7A–C), where we can figure out the SWD of epileptic absence seizure by the inset of Figure 7B.

In brief, thalamic input parameter *h*_*tc*_ and inhibitory strength *c*_*iny*_ can be referred to delayed control parameters of the onset of epileptic absence seizures characterized by the typical 2–4 Hz SWD as they are gradually increased or decreased respectively in the modified Taylor's model. In particular, disinhibition function can appropriately delay the onset of absence seizures in epilepsy, by dynamical bifurcation mechanisms study, where the fold of cycles and Hopf-type bifurcations play a key role in these dynamical transitions.

## Discussion and conclusion

In the previous study (Taylor and Baier, 2011, Taylor et al., 2013b), the investigations emphasized the potential interest of nonlinear neural field modeling of epileptic absence seizures characterized by periodic spike and slow-wave (SWD) complexes in some simplified neural field models. Additionally, underlying disinhibition mechanisms in a subnetwork module potentially stabilize the balance of electrical signals between neurons in the nervous system globally, the size of which can change the output of the neuron's signal strength within the module. However, these models shortcomings was exclusive of the cortical interneuron disinhibition mechanisms.

In the present study, already published consensus confirmation about: (i) the involvement of thalamus in the process of absence seizures in epilepsy; (ii) the depression of interneuron inhibition by another inhibitory interneuron effect in the cortical subnetwork have been introduced in the same class of macroscopic neural field models. This model includes two main features: an thalamic TC-RE circuit module by the activation of *GABA*_*A*_ and glutamatergic receptors that forms the thalamic function into cortical subnetwork, and an inhibitory feedback loop that bears two inhibitory neural populations by the activation of *GABA*_*A*_ and *GABA*_*B*_ receptors with different time scales, as suggested in Fan et al. (2015).

The modifications allow the model to display various dynamical behaviors including the saturated firings with different spatial scales, periodic spike and slow-wave discharges and simple oscillations. And also, it is found that the modified model can capture the 2–4Hz SWD and simple oscillations that represent the typical generalized epileptic wave seizures, i.e., absence seizures and clonic seizures, respectively. Moreover, we have investigated the onsets of epileptic SWD oscillations. It is shown that the disinhibition in the proposed model can modulate the interactions between the inhibitory input into PY population *c*_*iny*_ and the external inhibitory input *h*_*tc*_ on the thalamus, which can eventually lead to the delayed onsets of the epileptic SWD. More importantly, dynamical bifurcation mechanisms of these delayed onsets of SWD have been explored and clarified in detail, where the fold of cycles and Hopf-type bifurcations play a key role in these dynamical transitions.

Consideration of disinhibition mechanism between inhibitory interneuron populations in absence epilepsy suggests that the *GABA*_*B*_ modulation plays a key role in the process of absence seizures in epilepsy apart from the significance of *GABA*_*A*_ receptor in expression of absence seizures in epilepsy (Fritschy, 2008). The functional impact may be further compounded by the observation in the study of Caddick and Hosford (1996). Recent experiments have witnessed that there exist two inhibitory neural populations mainly mediated by *GABA*_*A*_ and *GABA*_*B*_ receptors that act as the fast and slow time scales of inhibition effects respectively, which certainly shape mutual inhibitory effect. This may indicate that absence seizure in epilepsy is correlated with a disturbance in inhibition from GABAergic neurons, which is a rational standpoint to complement in our study the underlying mechanisms in absence epilepsy. But here, insights into the neurophysiological mechanism underlying the balance/imbalance between these inhibitory processes of cortical inhibitory feedback module are ignored, instead the newly excitatory-inhibitory pathway intermediated by *GABA*_*A*_ receptor is selectively considered in the present study. Resultantly, the *GABA*_*A*_ modulation and indirect disinhibition function between inhibitory neural populations could lead to a delay onset of absence seizures in epilepsy in contrast to the results from Fan et al. (2015), though the mechanisms under the electrophysiologically observed epileptic SWD are very complex, and the exact cellular mechanisms underlying the SWD are still unknown. However, it is still initial stage to investigate functional effects of disinhibitory neural population on firing dynamics. Hence, we hope that our results can further improve the understanding of controlling epileptic absence seizures.

One of the assumptions of our study is the existence of thalamic dynamic input from other nonspecific projections or external noises in a neural field model, a good candidate for understanding mechanisms of epileptic seizure that can just roughly represent the activity of neuronal populations. This dynamic input control in our model is closely associated with the typical cortical SWD activity in a bidirectional modulation fashion, as well as with the work of Chen et al. (2015) and Taylor et al. (2015) without the interneuron disinhibition. And by comparison, the additional involvement of disinhibition can indeed delay the appearance of SWD pattern in thalamic dynamic input window dynamically. Furthermore, these observations are for the origin of the generalized SWD associated with absence epilepsy from the perspective of synthetic cortico-thalamic system.

In conclusion, this computational study indicates that the thalamic dynamic input and the enhanced *GABA*_*A*_ inhibition to excitatory neural population in absence epilepsy may give rise to the delay onset of seizures. This result highlights the functional role of the disinhibitory neural population in controlling absence seizures. Further investigations of the computational models for absence epilepsy should be based on the neural networks (Jirsa et al., 2014; El Houssaini et al., 2015; Fan et al., 2015; Khambhati et al., 2015; Taylor et al., 2015), which can well uncover some more detailed mechanisms of epileptic SWD as the disinhibitory neural population is introduced, and has potential guideline in the clinical treatment of absence epilepsy.

## Author contributions

SL, QW, and DF organized and wrote the paper.

### Conflict of interest statement

The authors declare that the research was conducted in the absence of any commercial or financial relationships that could be construed as a potential conflict of interest.
